# Common Cervicovaginal Microbial Supernatants Alter Cervical Epithelial Function: Mechanisms by Which *Lactobacillus crispatus* Contributes to Cervical Health

**DOI:** 10.3389/fmicb.2018.02181

**Published:** 2018-10-08

**Authors:** Lauren Anton, Luz-Jeannette Sierra, Ann DeVine, Guillermo Barila, Laura Heiser, Amy G. Brown, Michal A. Elovitz

**Affiliations:** Department of Obstetrics and Gynecology, Maternal and Child Health Research Center, Perelman School of Medicine at the University of Pennsylvania, Philadelphia, PA, United States

**Keywords:** cervix, *Lactobacillus crispatus*, *Lactobacillus iners*, *Gardnerella vaginalis*, epithelial barrier, inflammation, miRNA, preterm birth

## Abstract

Cervicovaginal (CV) microbiota is associated with vaginal health and disease in non-pregnant women. Recent studies in pregnant women suggest that specific CV microbes are associated with preterm birth (PTB). While the associations between CV microbiota and adverse outcomes have been demonstrated, the mechanisms regulating the associations remain unclear. As the CV space contains an epithelial barrier, we postulate that CV microbiota can alter the epithelial barrier function. We investigated the biological, molecular, and epigenetic effects of *Lactobacillus crispatus, Lactobacillus iners*, and *Gardnerella vaginalis* on the cervical epithelial barrier function and determined whether *L. crispatus* mitigates the effects of lipopolysaccharide (LPS) and *G. vaginalis* on the cervical epithelial barrier as a possible mechanism by which CV microbiota mitigates disease risk. Ectocervical and endocervical cells treated with *L. crispatus, L. iners*, and *G. vaginalis* bacteria-free supernatants alone or combined were used to measure cell permeability, adherens junction proteins, inflammatory mediators, and miRNAs. Ectocervical and endocervical permeability increased after *L. iners* and *G. vaginalis* exposure. Soluble epithelial cadherin increased after exposure to *L. iners* but not *G. vaginalis* or *L. crispatus*. A Luminex cytokine/chemokine panel revealed increased proinflammatory mediators in all three bacteria-free supernatants with *L. iners* and *G. vaginalis* having more diverse inflammatory effects. *L. iners* and *G. vaginalis* altered the expression of cervical-, microbial-, and inflammatory-associated miRNAs. *L. crispatus* mitigated the LPS or *G. vaginalis*-induced disruption of the cervical epithelial barrier and reversed the *G. vaginalis*-mediated increase in miRNA expression. *G. vaginalis* colonization of the CV space of a pregnant C57/B6 mouse resulted in 100% PTB. These findings demonstrate that *L. iners* and *G. vaginalis* alter the cervical epithelial barrier by regulating adherens junction proteins, cervical immune responses, and miRNA expressions. These results provide evidence that *L. crispatus* confers protection to the cervical epithelial barrier by mitigating LPS- or *G. vaginalis*-induced miRNAs associated with cervical remodeling, inflammation, and PTB. This study provides further evidence that the CV microbiota plays a role in cervical function by altering the cervical epithelial barrier and initiating PTB. Thus, targeting the CV microbiota and/or its effects on the cervical epithelium may be a potential therapeutic strategy to prevent PTB.

## Introduction

Preterm birth (PTB), defined as delivery at less than 37 weeks of gestation, remains the leading cause of perinatal morbidity and mortality. Of all live births reported in 2016, 9.8% were preterm deliveries. Babies born prematurely often face, not only neonatal, but also, lifelong medical complications leading to an estimated 26.2 billion dollars in annual medical and societal expenses in the United States alone (Behrman and Butler, 2007). Despite decades of research investigating the biological mechanisms contributing to the pathogenesis of PTB, the underlying cause(s) remains largely unknown. Due to this lack of understanding, even though the PTB rate has decreased slightly over the last decade (March of Dimes, Peristats, 2017: 12.8% in 2006 to 9.8% in 2016), very few therapeutic attempts to prevent or reduce PTB have been proven successful.

Spontaneous PTB (sPTB) is well recognized to be a complex syndrome as it has been associated with multiple disease mechanisms including inflammation and infection, stress, uteroplacental ischemia, and uterine overdistension among many others (Goldenberg et al., 2008). The most recognized of these theories is that an ascending infection from the vagina/cervix to the uterus causes an inflammatory response that contributes to the initiation of uterine contractions resulting in spontaneous preterm labor (Romero et al., 1994; Goldenberg et al., 2000; Chang et al., 2012; Hutchinson et al., 2014). However, multiple clinical trials targeting infection with antibiotic therapy failed to alter the PTB rate (Romero et al., 1993; Cox et al., 1996; Kenyon et al., 2001). Therefore, recent studies by our group have focused on alterations within the cervicovaginal (CV) space and their contributions to premature cervical remodeling as a primary, if not, initiating step in the onset of PTB (Gonzalez et al., 2009; Nold et al., 2012; Elovitz et al., 2014). Understanding the biological mechanisms regulating cervical remodeling is critical to developing therapies to treat or diagnose PTB.

Cervical remodeling is a natural continually occurring process over the course of gestation that is broken down loosely into four phases: softening, ripening, dilation, and postpartum repair (Word et al., 2007). A normal function of the cervix is essential to a successful pregnancy. The cervix has several critical functions including (1) maintenance of the integrity of the cervical epithelial barrier that lines the cervical lumen and acts to prevent microbial invasion and infection and (2) appropriate timing of cervical remodeling to prepare for delivery. Previous studies by our group and others have shown that many factors can promote a breakdown in the cervical epithelial barrier and initiate cervical remodeling through alterations in inflammation and infection (Nold et al., 2012), microRNAs (miRNAs) (Anton et al., 2017), biomechanical properties of the cervix (Yoshida et al., 2016; Barnum et al., 2017), and the CV metabolome (Ghartey et al., 2015, 2017) and microbiome (Romero et al., 2014a; Stout et al., 2017). Differentially expressed miRNAs in the CV space are associated with PTB (Elovitz et al., 2014), short gestation (Sanders et al., 2015), and disruption of the cervical epithelial barrier (Anton et al., 2017). While a link between altered miRNA expression and cervical cell function has been established (Anton et al., 2017), the regulation of miRNAs within the CV space remains unknown.

The human microbiome has recently become the focus of many studies, encouraged by the National Institutes of Health-funded Human Microbiome Project (NIH HMP Working Group et al., 2009), investigating the role of infection and inflammation in multiple disease states including inflammatory bowel disease, autoimmune disease, cancer, and asthma among many others (Cho and Blaser, 2012). With the development of non-culture characterization of bacterial communities through 16S ribosomal RNA (16S rRNA) sequencing (Conlan et al., 2012), the identification of specific microbiota inhabiting the human body, including the vagina/cervix, has become much more attainable. While it is well known that the *Lactobacillus* bacterial species are common inhabitants of the human vagina and are typically (although not always) indicative of a healthy vaginal space due to their production of lactic acid (and resulting low pH) (Redondo-Lopez et al., 1990), recent work by Ravel et al. (2011) and others have more comprehensively characterized the vaginal microbiota in both non-pregnant and pregnant women (Romero et al., 2014a,b; MacIntyre et al., 2015). In healthy asymptomatic non-pregnant women of reproductive age, this study reported the detection of an abundance of vaginal bacterial species that have been divided into at least six community state types (CSTs) (Ravel et al., 2011). While four of these CSTs are dominated by *Lactobacillus* spp. including *L. crispatus* (CST I), *L. gasseri* (CST II), *L. iners* (CST III), and *L. jensenii* (CST V), the other two CSTs (CST IV-A and IV-B) are composed of mostly anaerobic bacteria, *Gardnerella vaginalis* (*G. vaginalis*), *Mobiluncus, Atopobium vaginae, Bacteroides*, and *Prevotella*. These microbes are typically associated with unhealthy or dysbiotic vaginal states such as bacterial vaginosis (BV), HIV, and adverse obstetrical outcomes, including sPTB (Holst et al., 1994; Hashemi et al., 1999; Fredricks et al., 2007; Bretelle et al., 2015; Kindinger et al., 2016). The concept that the non-*Lactobacillus* dominated microbial communities confer risk is supported by multiple studies demonstrating an association between the presence of BV and sPTB (Hillier et al., 1995; Kimberlin and Andrews, 1998). However, clinical trials aimed at targeting BV have not shown alterations in PTB rates (Brocklehurst et al., 2013; Thinkhamrop et al., 2015). Traditionally, the diagnosis of BV has been made clinically without the use of 16S rRNA characterization of the microbial communities present in the CV space. In the last few years, investigations seeking to determine if microbial communities are associated with sPTB have been pursued. While these studies are limited in sample size, they have demonstrated some associations between the vaginal microbiota and sPTB (DiGiulio et al., 2015; Callahan et al., 2017; Stout et al., 2017). In these previous studies, lower *Lactobacillus* and higher *Gardnerella* abundance was found to be associated with sPTB in a low-risk cohort of women (DiGiulio et al., 2015; Callahan et al., 2017). In another study, decreased vaginal microbial richness and diversity in the first and second trimester of pregnancy was found to be associated with PTB (Stout et al., 2017). However, these studies do not address the possible molecular mechanisms by which specific bacterial species, common to the CV space, may contribute to sPTB.

Therefore, the objective of this study was to determine if specific CV bacterial species have the ability to alter the cervical epithelial barrier, differentially modify the host cervical epithelial immune response and/or alter the molecular and epigenetic pathways that regulate the cervical epithelial barrier. Based on previous studies, we chose to investigate three different bacterial species known to be associated with a spectrum of effects within the CV space. These are (1) *L. crispatus*, from CST I, which is the most predominant bacteria in the human vagina and is normally considered a representative of a healthy CV space (Redondo-Lopez et al., 1990; Atassi et al., 2006; Spurbeck and Arvidson, 2011); (2) *L. iners*, from CST III, which is an ambiguous intermediate bacterial species that has been considered a commensal CV bacteria as well as having an association with vaginal dysbiosis (Kindinger et al., 2017; Petrova et al., 2017; Vaneechoutte, 2017), HIV, and STDs (Hummelen et al., 2010; Borgdorff et al., 2014); and (3) *G. vaginalis*, from CST IV, which is a bacterial species most recognized for its association with BV (Cook et al., 1989). For this study, we investigated the effects of *L. crispatus, L. iners*, and *G. vaginalis* bacteria-free supernatants on cervical epithelial cell permeability to determine if these microbes have the ability to alter the cervical epithelial cell barrier. Additionally, we focused on the molecular and epigenetic mechanisms contributing to the breakdown of the cervical epithelial cell barrier by investigating the effects of these three bacteria-free supernatants on cell-to-cell adhesion, inflammation, and miRNA expression. We also created a pregnant mouse model with a *G. vaginalis*-colonized CV space to determine if colonization of this bacterial species could induce sPTB. We hypothesize that *L. crispatus*, a normal inhabitant of a healthy CV space, will protect and strengthen the cervical epithelial barrier to prevent the invasion of pathogens, while *L. iners* and *G. vaginalis*, bacteria associated with increased risk of BV, STD, or HIV acquisition, will disrupt the integrity of the cervical epithelial barrier through the regulation of cell adhesion, inflammatory, and miRNA mediators that contribute to premature cervical remodeling and PTB.

## Materials and Methods

### Cell Culture

Ectocervical (Ect/E6E7, AATC# CRL-2614) (Ecto) and endocervical (End1/E6E7, AATC# CRL-2615) (Endo) cell lines (American Type Culture Collection, Bethesda, MD, United States) were cultured in keratinocyte serum-free medium (K-SFM) supplemented with 0.1 ng/ml epidermal growth factor and 50 μg/ml bovine pituitary extract (ScienCell Research Laboratories, Carlsbad, CA, United States), 100 U/mL penicillin, and 100 μg/mL of streptomycin at 37°C in a 5% CO_2_ humidified incubator.

### Bacterial Strains and Preparation of Bacteria-Free Supernatants

*Lactobacillus crispatus* (*L. crispatus*) is a clinical human isolate that was collected, sequenced, identified, and gifted to us by Jacques Ravel at the University of Maryland. *Lactobacillus iners* (*L. iners*, ATCC 55195) and *Gardnerella vaginalis* (*G. vaginalis*, ATCC 14018) (Gardner and Dukes, 1959; Oshima et al., 2015) were purchased from American Type Culture Collection (ATCC, Rockville, MD, United States). All bacteria, their specific strains, and sites of origin are listed in **Table 1**. *L. crispatus* was grown in NYCIII media with 10% horse serum at 37°C in an anaerobic jar in a 5% anaerobic CO_2_ incubator for 7 days. *L. iners* was grown in Tryptic Soy Broth (TSB) (Becton, Dickson and Company, Sparks, MD, United States) with 5% defibrillated sheep blood (Rockland Immunochemicals, Limerick, PA, United States) at 37°C in an anaerobic jar in a 5% anaerobic CO_2_ incubator for 7 days. *G. vaginalis* was grown in Tryptic Soy Broth with 5% defibrillated rabbit blood (Rockland Immunochemicals, Limerick, PA, United States) at 37°C in an anaerobic jar in a 5% anaerobic CO_2_ incubator for 7 days. The cultures were centrifuged two times for 10 min each at 2,500 rpm at 4°C to remove the bacteria. The resulting supernatants were sterile-filtered through a 0.22 μM membrane filter (EMD Millipore, Darmstadt, Germany) to remove any remaining bacterial components or debris. The bacteria-free supernatants and each bacterium’s specific growth media (negative control to determine the baseline measurements of background growth media) were then used for *in vitro* cell culture experiments.

**Table 1:** Bacterial species used in study experiments.

Bacterial species	Source	Strain	Origin
*L. crispatus*	Jacques Ravel, University of Maryland	Unknown	Clinical isolate from a 38-year-old Caucasian female
*L. iners*	ATCC 55195	AB107	Patient with bacterial vaginosis
*G. vaginalis*	ATCC 14018	JCM11026	Isolated from the vaginal space of women with non-specific vaginitis in 1959, single species sequenced in 2015


The consistency of bacterial growth from week to week (and between experiments) was assessed by consistent optical density readings in the 450–600 nm range. Additionally, colony-forming unit assays (CFUs) were performed after 72 h of growth to determine the amount of bacteria per milliliter from which the bacteria-free supernatants were collected (**Supplementary Table 1**).

### Ectocervical Cells Treatments

Ectocervical cells were plated at 2.0 × 10^5^ cells/well in six-well plates. The next day, the cells were treated with 10% (v/v) bacteria-free supernatants in K-SFM cell growth media. For cells treated with bacteria-free supernatants from *L. crispatus*, the K-SFM medium was supplemented with 50 mM HEPES and sodium bicarbonate (3000 mg/L total concentration) to bring the pH of the medium up to a physiological level (7.2) as *L. crispatus* bacteria produces high amounts of lactic acid during growth. Without pH adjustments, even at lower volume per volume percentages, the cells did not survive. Ectocervical cells were treated with *L. crispatus* (*n* = 3), *L. iners* (*n* = 3), and *G. vaginalis* (*n* = 3) bacteria-free supernatants for 48 h. In additional experiments, ectocervical cells were dually treated with bacteria-free supernatants of *L. crispatus* and *G. vaginalis* where the cells were exposed to *L. crispatus* supernatants (5% v/v) on day 1 followed by *G. vaginalis* supernatants (5% v/v) on day 2 or vice versa for 24–48 h (*n* = 6–9). For all experiments, cells were also treated with the *L. crispatus, L. iners*, or *G. vaginalis* bacterial growth media alone as a negative control to determine any baseline effects of the growth media on the outcome of interest. At the end of each experiment, cell culture media were collected for ELISA assays and/or the cells were collected in TRIzol for RNA extraction.

### Cell Permeability Experiments

Ectocervical and endocervical cell permeability was determined using an In Vitro Vascular Permeability Assay kit (Millipore, Bedford, MA, United States). Briefly, ectocervical and endocervical cells were plated at 1.0 × 10^6^ cells/ml into 24-well hanging cell culture inserts, which contain 1 μm pores with a transparent polyethylene terephthalate (PET) membrane precoated with collagen. The cells were treated with 10% (v/v) bacteria-free supernatants from *L. crispatus* (*n* = 6), *L. iners* (*n* = 6), and *G. vaginalis* (*n* = 9) for 48 h. In additional experiments, the ectocervical cells were treated with the three bacteria-free supernatants as described above and 24 h later, the cells were exposed to the bacterial by-product, lipopolysaccharide (LPS, 25 μg/ml) (*Escherichia coli* 055:B5, Sigma, St. Louis, MO, United States) for additional 24 h (*n* = 6–9). In a third set of experiments, ectocervical cells were dually treated with supernatants from *L. crispatus* and *G. vaginalis* (as described above) for 24–48 h (*n* = 9). For all experiments, cells were also treated with the *L. crispatus, L. iners*, or *G. vaginalis* bacterial growth media alone as a negative control to determine any baseline effects of the growth media on cell permeability. After 72 h of total growth time, the media were removed and phenol red-free K-SFM media (ScienCell Research Laboratories, Carlsbad, CA, United States) containing FITC-Dextran was added to the top of the insert. The movement of FITC-Dextran from the top insert to the bottom was measured after 2 h by using a fluorescent plate reader at 485 and 535 nm, excitation and emission, respectively.

### ELISAs

Ectocervical cells were cultured in six-well plates and treated with bacteria-free supernatants as stated above. Soluble E-cadherin (sECAD), IL-6, and IL-8 were measured in ectocervical cell culture media after 48 h of treatment with bacteria-free supernatants or bacteria growth media from *L. crispatus, L. iners*, and *G. vaginalis* alone. The levels of sECAD, IL-6, and IL-8 were measured by ligand-specific commercially available ELISA kits that use a quantitative sandwich enzyme immunoassay technique using regents from R&D Systems (Minneapolis, MN, United States).

### Luminex Assay

Ectocervical cells were cultured and treated with bacteria-free supernatants as stated above. A 41-plex human cytokine/chemokine magnetic bead Luminex panel I (HCYTMAG-60K-PX41, EMD Millipore, Billerica, MA, United States) was run on ectocervical cell culture media after 48 h of treatment with *L. crispatus* (*n* = 3), *L. iners* (*n* = 3), and *G. vaginalis* (*n* = 3) bacteria-free supernatants. The samples were run in duplicate, per the manufacturer’s protocol on the FLEXMAP3D Luminex platform (Luminex, Austin, TX, United States). Data were analyzed using a standard curve generated by a five-parameter logistic (5PL) curve fit using Xponent 4.2 software (Luminex).

### miRNA Isolation From Ectocervical Cells

Following the bacteria-free supernatant treatments in six-well plates described above, ectocervical cells were washed in sterile PBS and collected in TRIzol (Invitrogen, Thermo Fisher Scientific) and underwent phenol–chloroform extraction. The resulting aqueous phase was further column-purified with the miRNeasy kit (Qiagen, Hilden, Germany) according to the manufacturer’s protocol for total RNA isolation including small RNAs. RNA concentration was determined via a NanoDrop 2000 Spectrophotometer (NanoDrop^TM^, Rockland, DE, United States) prior to the generation of cDNA.

### cDNA Generation and qPCR

cDNA was generated from 1 μg of isolated mRNA from ectocervical cells using the miScript II Reverse Transcription kit (miRNA) (Qiagen) for SYBR Green. qPCR was performed on the QuantStudio 7 Flex Real-Time PCR System (Applied Biosystems, Life Technologies) using the miScript SYBR Green PCR kit (Qiagen) according to the manufacturers’ protocol. The ΔΔCT method was used for relative expression quantification using the QuantStudio Real-time PCR software v1.1 (Applied Biosystems, Life Technologies). The endogenous reference gene RNU6B was used for miRNA quantification. All miRNA primer sets were purchased from Qiagen: miR-143 (MS00003514), miR-145 (MS00003528), miR-193b (MS00031549), miR-146a (MS00003535), miR-223 (MS00003871), miR-148b (MS00031458), miR-15a (MS00003178), miR-21(MS00009079), miR-142 (MS00031451), miR-494 (MS00033754), miR-30e (MS00009401), and RNU6B (MS0001400).

### Animals

C57/B6 timed-pregnant mice were purchased from Charles River Laboratories (Wilmington, MA, United States). We considered E0 as mating day and E1 was determined based on the presence of copulatory plug. Animals were shipped on day 10 after mating and housed individually in our facilities. These animals were acclimated for 4 days before experiments were performed. All the experiments were performed in accordance with the National Institutes of Health Guidelines on Laboratory Animals and with approval from the University of Pennsylvania’s Institutional Animal Care and Use Committee (IACUC #:805513).

### Cervicovaginal Colonization With *G. vaginalis*

A pregnant mouse model of *G*. *vaginalis* colonization was created in the following manner. Timed-pregnant, C57/B6 mice, were anesthetized with isoflurane on embryonic day 14 (E14) and five CV lavages were performed with 100 μL of sterile PBS prior to control treatment or bacterial inoculation. Bacterial doses were determined using published data in a non-pregnant mouse model (Gilbert et al., 2013), and then recapitulating similar *G. vaginalis* loads in pregnant mice. Two animal trials were performed to determine (1) PTB outcome and (2) CV colonization by *G. vaginalis*. For the first trial, on E14, E15, and E16, the animals received an intravaginal inoculation of *G. vaginalis* (*n* = 9) by inserting a sterile pipette tip and injecting 20 μL of 5 × 10^9^ CFU/animal or sugar water (10% fructose, 10% maltose, 10% glucose, 10% dextrose) (*n* = 6) into the mouse vagina. These time points were chosen to mimic a change in the CV microflora at mid-pregnancy. Immediately post-inoculation, each animal was positioned in dorsal decubitus under isoflurane anesthesia for 3 min and 100% pure petroleum jelly (Vaseline, Unilever USA) was added to the cervix with a sterile swab to ensure that the inoculum would remain within the CV space. The animals were observed twice daily for PTB until E19 (term). In the second animal trial, on E14 the animals received an intravaginal inoculation of *G. vaginalis* (*n* = 5) of 5 × 10^9^ CFU/animal or sugar water (*n* = 4). Twenty-four hours later, the animals were sacrificed and CV fluid (CVF) was collected to assess for *G. vaginalis* colonization of the CV space. The CVF was collected by gently rinsing the CV space (pipetting in and out seven times) with 100 μL of sterile PBS. The washes were pooled together into one sterile tube for each dam.

### Genomic DNA Isolation and QPCR

Genomic DNA (gDNA) was isolated and purified from the CVF with the ZR fecal MiniPrep DNA extraction kit (Zymo Research, Irvine, CA, United States). To quantify the amount of *G. vaginalis* gDNA, we used a TaqMan 16S specific probe to this bacterium (Applied Biosystems, Foster City, CA, United States). gDNA from the CVF was quantified by QPCR to determine tissue-specific colonization. A standard curve was created from serially diluted gDNA from *G. vaginalis* to quantify the amplification. This standard curve was used for relative quantification of *G. vaginalis* abundance using the 7900HT Real-Time PCR System (Applied Biosystems). The results were analyzed using the RQ manager software v2.4 (Applied Biosystems). In TaqMan QPCR assays, the relative abundance of the target of interest was divided by the relative abundance of pan-bacterial 16S rRNA in each sample to generate a standardized abundance for the target transcript of interest.

### Statistical Analysis

Statistical analyses were performed for all experiments with the GraphPad Prism Software (Version 7.0, San Diego, CA, United States). For data that were normally distributed, one-way analysis of variance (ANOVA) was performed. If statistical significance was reached (*p* < 0.05), then pair-wise comparison with a Tukey *post hoc* test was performed for data that had similar variances. However, if the variances between groups were significantly different, then a Sidak *post hoc* test was performed. If data were not normally distributed, then the non-parametric Kruskal–Wallis test was used and pairwise comparison was performed using a Dunn’s multiple comparison test. To compare two groups (animal data), unpaired *t*-tests were performed. If the data did not have equal variances, then an unpaired *t*-test with Welch’s correction was done.

## Results

### *L. iners* and *G. vaginalis* Increase Ectocervical and Endocervical Cell Permeability

**FIGURE 1:**
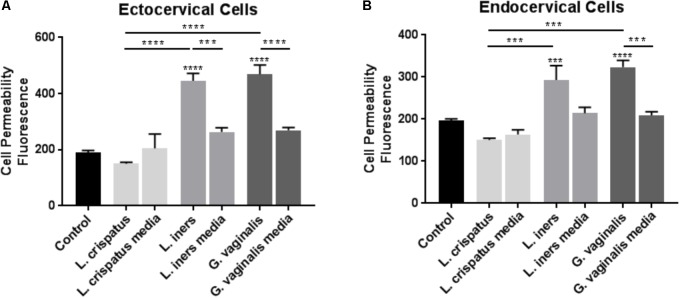
Cell permeability is increased in ectocervical and endocervical cells after exposure to *L. iners* and *G. vaginalis* but not *L. crispatus* bacteria-free supernatants. Cell permeability was measured in ectocervical cells **(A)** and endocervical cells **(B)** after a 48 h exposure to bacteria-free supernatants (10% v/v) from *L. crispatus, L. iners*, and *G. vaginalis* compared to non-treated control cells. Bacterial growth media alone acted as a negative control for the three bacteria-free supernatants tested. Cell permeability is expressed as fluorescence OD measurements from a fluorescent plate reader and is indicative of the movement of FITC-dextran from the top to the bottom insert of a transwell chamber system. Values are mean ± SEM. Asterisks over the individual bars represent comparisons to control; asterisks over solid lines represent comparisons between treatment groups. ^∗^*p* < 0.05, ^∗∗^*p* < 0.01, ^∗∗∗^*p* < 0.001, ^∗∗∗∗^*p* < 0.0001.

To determine if common vaginal bacterial species release factors within the CV space that have the ability to alter the cervical epithelial barrier, we performed cell permeability assays in ectocervical and endocervical cells after treatment with bacteria-free supernatants. Ectocervical cell permeability (**Figure 1A**) was unchanged after treatment with *L. crispatus* supernatants (*p* = 0.8660), while *L. iners* (*p* < 0.0001), and *G. vaginalis* (*p* < 0.0001) supernatants significantly increased cell permeability. A similar effect was seen in endocervical cells (**Figure 1B**), as cell permeability was unchanged in the presence of bacteria-free supernatants from *L. crispatus* (*p* = 0.2942) and increased by *L. iners* (*p* = 0.0006), and *G. vaginalis* (*p* < 0.0001). There was no significant effect of the bacterial growth media alone on cell permeability of ectocervical or endocervical cells for any of the bacteria tested. Additionally, the increase in cell permeability after treatment with bacteria-free supernatants from both *L. iners* (*p* = 0.0002) and *G. vaginalis* (*p* < 0.0001) in ectocervical cells and *G. vaginalis* (*p* < 0.0001) only in endocervical cells was significantly higher than the bacterial growth media alone.

### *L. iners* Alters Ectocervical Cell Adhesion by Cleaving E-cadherin

**FIGURE 2:**
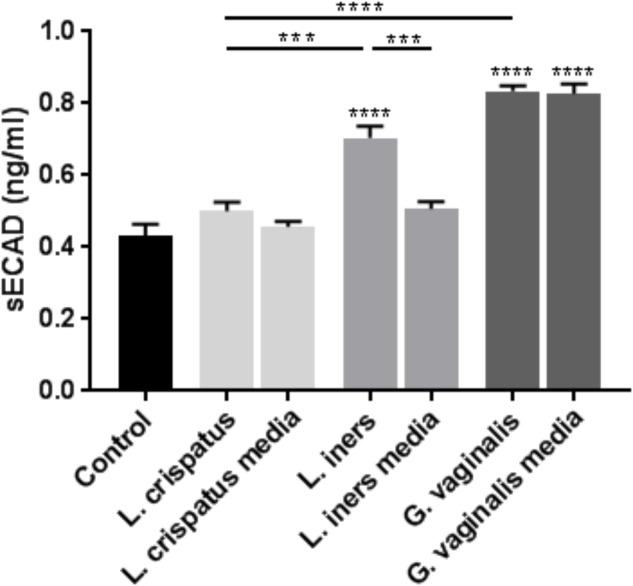
*Lactobacillus iners* and *G. vaginalis* but not *L. crispatus* bacteria-free supernatants increase epithelial-cadherin cleavage in ectocervical cells. Soluble epithelial-cadherin (sECAD) levels were measured after ectocervical cells were exposed to *L. crispatus, L. iners*, and *G. vaginalis* bacteria-free supernatants (10% v/v) for 48 h. Bacterial growth media alone acted as a negative control for the three bacteria-free supernatants tested. sECAD was measured in ectocervical cell culture supernatants using a commercially available sandwich ELISA. Values are mean ± SEM. Asterisks over the individual bars represent comparisons to control; asterisks over solid lines represent comparisons between treatment groups. ^∗^*p* < 0.05, ^∗∗^*p* < 0.01, ^∗∗∗^*p* < 0.001, ^∗∗∗∗^*p* < 0.0001.

As we observed similar significant changes in cell permeability with both ectocervical and endocervical cells, we chose to use ectocervical cells as a model system in all of the following experiments that were performed to further elucidate the mechanisms regulating the observed changes in cervical epithelial barrier function. We sought to investigate if bacteria-free supernatants were able to alter the adherens junction complex that plays a critical role in the epithelial cell-to-cell adhesion needed to maintain the cervical epithelial barrier. As we have previously shown that cleavage of E-cadherin can act as a biomarker for reduced adherens junction and cervical epithelial barrier integrity (Nold et al., 2012; Anton et al., 2017), we measured sECAD levels after bacteria-free supernatant exposure in ectocervical cells. In the presence of *L. crispatus* bacteria-free supernatants, sECAD was unchanged; however, treatment with *L. iners* (*p* < 0.0001) and *G. vaginalis* (*p* < 0.0001) supernatants significantly increased sECAD concentration when compared to non-treated cells (control) (**Figure 2**). sECAD concentration was unchanged by both *L. crispatus* and *L. iners* growth media; however, there was a significant increase in sECAD after exposure to *G. vaginalis* growth media (*p* < 0.0001). Therefore, the elevated sECAD concentration measured after exposure to *G. vaginalis* bacteria-free supernatants was due to an effect of the growth media and not the secreted factors produced by the bacteria. sECAD was significantly higher after exposure to *L. iners* bacterial supernatant when compared to *L. iners* growth media alone (*p* = 0.0009) while *G. vaginalis* bacteria-free supernatant versus *G. vaginalis* growth media were not significantly different.

### Bacteria-Free Supernatants Alter Ectocervical Cell Inflammatory Mediators

**FIGURE 3:**
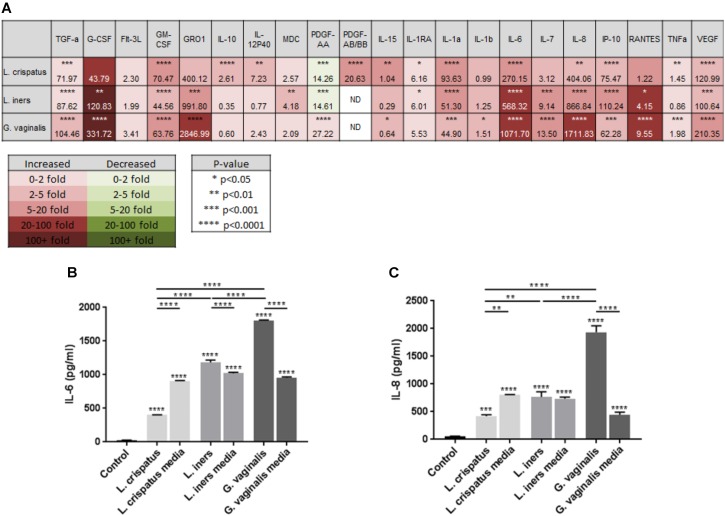
*Lactobacillus crispatus, L. iners*, and *G. vaginalis* bacteria-free supernatants differentially alter the host epithelial immune response in ectocervical cells. Immune cytokines/chemokines released from ectocervical cells after exposure to *L. crispatus, L. iners*, and *G. vaginalis* bacteria-free supernatants (10% v/v) for 48 h were measured by Luminex **(A)**. Heat map depicts fold change by color, *p*-value by asterisks, and concentration (pg/ml) of each analyte by the number value present in each corresponding box. Validation of Luminex findings were performed for IL-6 **(B)** and IL-8 **(C)** in ectocervical cells exposed to *L. crispatus, L. iners*, and *G. vaginalis* bacteria-free supernatants or bacterial growth media alone. IL-6 and IL-8 were measured in ectocervical cell culture supernatants using a commercially available sandwich ELISA. Values are mean ± SEM. Asterisks over the individual bars represent comparisons to control; asterisks over solid lines represent comparisons between treatment groups. ^∗^*p* < 0.05, ^∗∗^*p* < 0.01, ^∗∗∗^*p* < 0.001, ^∗∗∗∗^*p* < 0.0001.

As we have previously shown that the activation of an inflammatory response by the bacterial by-product LPS can initiate cervical epithelial breakdown (Nold et al., 2012), we wanted to determine if bacteria within the CV space have the ability to release mediators that can alter the host epithelial immune response. Therefore, we performed a discovery-based multiplex Luminex assay to assess the immune profile of ectocervical cells after exposure to *L. crispatus, L. iners*, and *G. vaginalis* bacteria-free supernatants. Of the 41 cytokines/chemokines present on the Luminex assay, 21 were found to be expressed by ectocervical cells (**Figure 3A**). A list of those cytokines/chemokines included in the Luminex assay that were not detectable in either non-treated (control) nor bacteria-free supernatant-treated ectocervical cells can be found in **Supplementary Table 2**. Overall, the results of the Luminex assay revealed a significantly varied and diverse immune profile between non-treated (control) ectocervical cells and those exposed to bacteria-free supernatants. After setting an arbitrary cutoff and focusing only on those cytokines/chemokines exhibiting greater than a five-fold change with a significant *p*-value <0.05, there were five cytokines/chemokines increased by *L. crispatus*, eight cytokines/chemokines increased by *L. iners*, and eight cytokines/chemokines increased by *G. vaginalis* (**Figure 3A**). Assuming that these cytokines/chemokines are acting in their traditional roles, out of the three bacteria-free supernatants tested, *L. crispatus* was the only bacterial supernatant to significantly increase the classical anti-inflammatory cytokine, IL-10 (*p* < 0.0001) (**Figure 3A**).

To validate the results of the Luminex assay and to assess any background results due to the bacterial growth media alone, we measured the classical proinflammatory cytokines known to be highly produced by ectocervical and endocervical cells (Fichorova and Anderson, 1999), IL-6 and IL-8, by performing ELISAs. The production of ectocervical IL-6 and IL-8 were significantly increased (**Figures 3B,C**, *p* < 0.001) after treatment with bacteria-free supernatants from *L. crispatus, L. iners*, and *G. vaginalis*. IL-6 and IL-8 concentrations were also significantly upregulated by *L. crispatus, L. iners*, and *G. vaginalis* growth media (*p* < 0.001). IL-6 and IL-8 levels were significantly decreased after exposure to *L. crispatus* bacteria-free supernatants when compared to its growth media (*p* < 0.01). On the contrary, both cytokines were increased after exposure to *G. vaginalis* bacteria supernatants versus *G. vaginalis* growth media (*p* < 0.0001). For *L. iners*, IL-6 expression was significantly increased after bacteria-free supernatant exposure when compared to growth media alone (*p* < 0.0001); however, IL-8 expression was unchanged between the two groups. Therefore, the elevated IL-6 and IL-8 concentration measured after exposure to *L. iners* bacteria-free supernatants was due to an effect of the growth media and not the secreted factors produced by the bacteria.

### *L. iners* and *G. vaginalis* Alter the miRNA Profile of Ectocervical Cells

**FIGURE 4:**
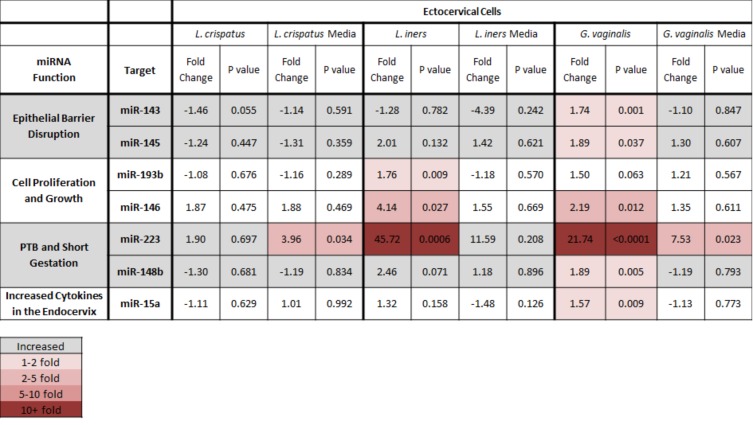
*Lactobacillus crispatus, L. iners*, and *G. vaginalis* bacteria-free supernatants differentially alter the miRNA expression profile of ectocervical cells. miRNA expression was measured by QPCR in ectocervical cells after exposure to *L. crispatus, L. iners*, and *G. vaginalis* bacteria-free supernatants (10% v/v) for 48 h. Bacterial growth media alone acted as a negative control for the three bacteria-free supernatants tested. The ΔΔCT method was used for relative expression quantification using the endogenous reference gene RNU6B. Modified heat map shows fold change versus control (non-treated cells) and corresponding *p*-values. Color distinguishes level of fold change. Values are mean ± SEM.

As alterations in miRNA expression have been shown to play a role in cervical epithelial cell function (Anton et al., 2017), we assessed if *L. crispatus, L. iners*, and *G. vaginalis* bacteria-free supernatants have the ability to alter specific miRNAs in ectocervical cells. Eleven miRNAs were chosen for further investigation based on previously published studies showing an association with sPTB, shorter gestation, and breakdown of the cervical epithelial barrier (Hassan et al., 2010; Sanders et al., 2015; Sanders et al., 2017). Ectocervical cell exposure to *L. iners* or *G. vaginalis* bacteria-free supernatants significantly increased miR-143, miR-145, miR-193b, miR-146, miR-223, miR-148b, and miR-15a expression (**Figure 4**, *p* < 0.05). *L. crispatus* bacteria-free supernatants had no effect on the expression of any of the miRNAs measured (**Figure 4**). With the exception of miR-223 (seen with *L. crispatus* and *G. vaginalis* media), no significant changes in miRNA expression were seen with *L. crispatus, L. iners*, or *G. vaginalis* growth media alone. miRNAs showing no significant changes in expression after exposure to any of the three bacteria-free supernatants tested are listed in **Supplementary Table 3**.

### *L. crispatus* Is Protective Against Inflammation-Mediated Increases in Ectocervical Cell Permeability

**FIGURE 5:**
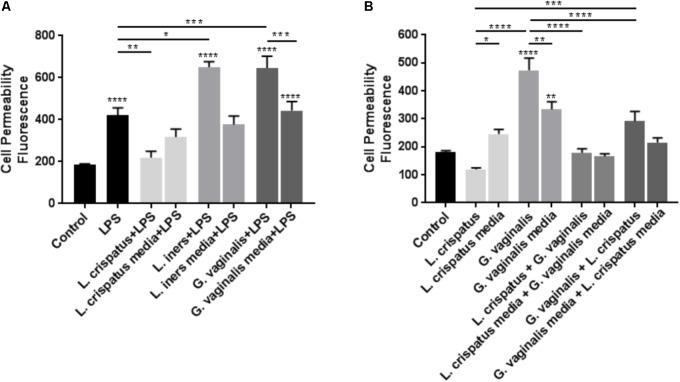
*Lactobacillus crispatus* bacteria-free supernatants mitigate the LPS- or *G. vaginalis*-induced increases in cell permeability in ectocervical cells. **(A)** Cell permeability was measured in ectocervical cells after exposure to bacterial free supernatants (10% v/v) from *L. crispatus, L. iners*, and *G. vaginalis* for 24 h followed by LPS (25 μg/ml) exposure for additional 24 h. Bacterial growth media containing LPS acted as a negative control for the three bacteria-free supernatants tested. **(B)** Cell permeability was measured in ectocervical cells after exposure to bacterial free supernatants from *L. crispatus* and *G. vaginalis* alone or in combination. Ectocervical cells were exposed to *L. crispatus* supernatants (5% v/v) on day 1 followed by *G. vaginalis* supernatants (5% v/v) on day 2 or vice versa for 24–48 h. Bacterial growth media alone acted as a negative control for the three bacteria-free supernatants tested. Cell permeability is expressed as fluorescence OD measurements from a fluorescent plate reader and is indicative of the movement of FITC-dextran from the top to the bottom insert of a transwell chamber system. Values are mean ± SEM. Asterisks over the individual bars represent comparisons to control; asterisks over solid lines represent comparisons between treatment groups. ^∗^*p* < 0.05, ^∗∗^*p* < 0.01, ^∗∗∗^*p* < 0.001, ^∗∗∗∗^*p* < 0.0001.

As we have shown that bacteria-free supernatants have the ability to alter cervical cell permeability, we wanted to determine if these supernatants could augment or mitigate cervical cell permeability in the presence of a general gram-negative *E. coli*-activated immune response. Previous studies conducted by our laboratory have shown that LPS has the ability to initiate PTB in an animal model (Elovitz et al., 2003) and that LPS exposure results in the breakdown of the cervical epithelial barrier (Nold et al., 2012). Therefore, we treated ectocervical cells with *L. crispatus, L. iners*, or *G. vaginalis* bacteria-free supernatants to simulate a CV space dominated by one of the three common vaginal bacterial species and 24 h later treated them with LPS to stimulate a general bacteria-induced immune response. These studies were designed to mimic the clinical scenario of women who have *L. crispatus*-, *L. iners*-, or *G. vaginalis*-dominated CV spaces and secondarily have a CV infection resulting in localized inflammation. In ectocervical cells (**Figure 5A**), as we have shown previously (Nold et al., 2012), LPS alone significantly increased cell permeability (*p* < 0.0001 vs. control). In the presence of *L. crispatus* bacteria-free supernatants, the LPS-induced increase in cell permeability was significantly inhibited (*p* = 0.0079 vs. LPS alone). However, in the presence of both *L. iners* (*p* = 0.0283 vs. LPS alone) and *G. vaginalis* (*p* = 0.0005 vs. LPS alone) bacteria-free supernatants, the LPS-induced increase in cell permeability was significantly enhanced. Following exposure to *L. crispatus, L. iners*, and *G. vaginalis* bacteria growth media containing LPS, ectocervical cell permeability was not significantly different than with LPS alone.

### *L. crispatus* Mitigates *G. vaginalis*-Induced Increases in Ectocervical Cell Permeability

As *L. crispatus* has a protective effect against inflammation-mediated cell permeability, we investigated if *L. crispatus* bacteria-free supernatants have the ability to mitigate the increased cell permeability observed in the presence of *G. vaginalis*. Exposure of ectocervical cells to *L. crispatus* bacteria-free supernatants followed by *G. vaginalis* bacteria-free supernatants resulted in a significant reduction in the *G. vaginalis*-induced increase in cell permeability (**Figure 5B**, *p* < 0.0001 vs. *G. vaginalis* alone). Additionally, exposure of ectocervical cells to *G. vaginalis* bacteria-free supernatants first followed by a secondary exposure to *L. crispatus* bacteria-free supernatants resulted in a similar significant reduction in the *G. vaginalis*-induced increase in cell permeability (**Figure 5B**, *p* < 0.0001 vs. *G. vaginalis* alone). The dual exposure to *L. crispatus* and *G. vaginalis* bacterial growth media had no effect on cell permeability independent of the order of exposure.

### *L. crispatus* Mitigates *G. vaginalis*-Induced Increases in Ectocervical Cell miRNA Expression

**FIGURE 6:**
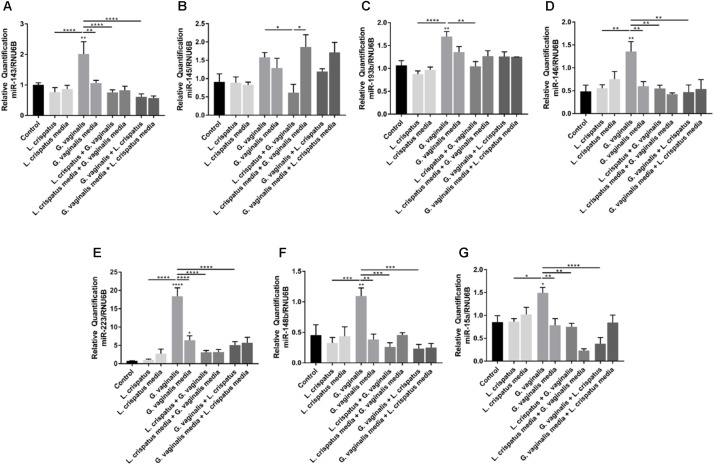
*Lactobacillus crispatus* bacteria-free supernatants mitigate the *G. vaginalis*-induced miRNA expression profile in ectocervical cells. miRNA expression **(A–G)** was measured by QPCR in ectocervical cells after exposure to *L. crispatus* supernatants (5% v/v) on day 1 followed by *G. vaginalis* supernatants (5% v/v) on day 2 or vice versa for 24–48 h. Bacterial growth media alone acted as a negative control for the three bacteria-free supernatants tested. The ΔΔCT method was used for relative expression quantification using the endogenous reference gene RNU6B. Values are mean ± SEM. Asterisks over the individual bars represent comparisons to control; asterisks over solid lines represent comparisons between treatment groups. ^∗^*p* < 0.05, ^∗∗^*p* < 0.01, ^∗∗∗^*p* < 0.001, ^∗∗∗∗^*p* < 0.0001.

Given the protective effects of *L. crispatus* bacteria-free supernatants against the increases in cell permeability seen with the *G. vaginalis* bacterial supernatants, we investigated if a reduction in the *G. vaginalis*-induced miRNA expression could be a potential mechanism. We investigated the effects of the dual exposure to *L. crispatus* and *G. vaginalis* bacteria-free supernatants in ectocervical cells on the expression of only those miRNAs that were found to be increased after *G. vaginalis* treatment including miR-143, miR-145, miR-193b, miR-146, miR-223, miR-148b, and miR-15a (**Figures 6A–G**). *G. vaginalis* bacteria-free supernatant-induced increases in miR-143, miR-146, miR-223, miR-148b, and miR-15a were all significantly reduced in the presence of *L. crispatus* bacteria-free supernatant independent of the order of supernatant exposure (**Figures 6A–G**, *p* < 0.01). *G. vaginalis* bacteria-free supernatant induced increases in miR-145 (**Figure 6B**) and miR-193b (**Figure 6C**) were only significantly reduced when the ectocervical cells were exposed to *L. crispatus* first followed by *G. vaginalis* but not the opposite. *L. crispatus* and *G. vaginalis* growth media alone or in combination had no effect on miRNA expression for any of the miRNAs measured.

### Intravaginal Inoculation of *G. vaginalis* Successfully Colonizes the CV Space and Results in PTB

**FIGURE 7:**
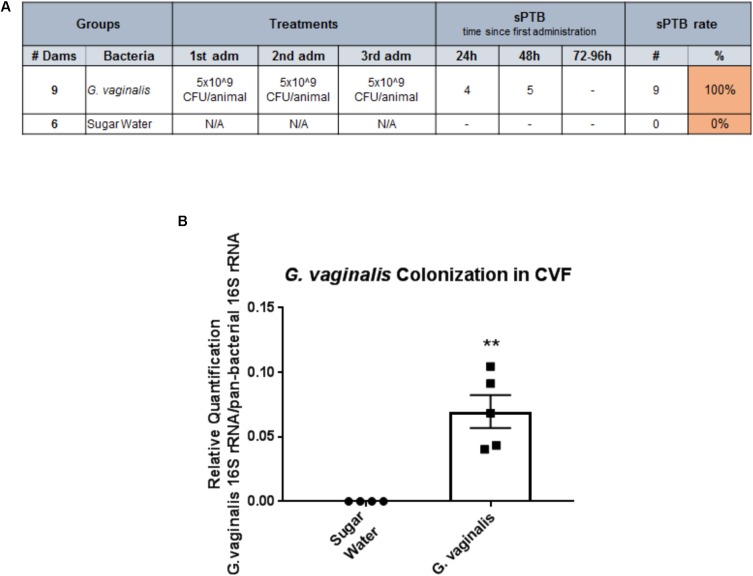
Intravaginal inoculation of *G. vaginalis* results in PTB and colonization of the cervicovaginal space. **(A)** *G. vaginalis* (5 × 10^9^ CFU/animal) was inoculated intravaginally into timed pregnant C57/B6 mice on embryonic day E13, E14, and E15. Sugar water inoculated animals acted as controls. Animals were observed for PTB daily until term. Table shows the dose of *G. vaginalis* inoculation at each administration, the number of dams delivering preterm on each day following the first inoculation, and the PTB percentage. **(B)**. *G. vaginalis* (5 × 10^9^ CFU/animal) was inoculated intravaginally into timed pregnant C57/B6 mice on E13. Animals were sacrificed 24 h after inoculation. Cervicovaginal fluid (CVF) was collected for gDNA isolation and measurement of the 16S gene of *G. vaginalis* by QPCR. Sugar water inoculated animals acted as controls. Values are mean ± SEM. ^∗∗^*p* < 0.01.

As we have shown that *G. vaginalis* bacteria-free supernatants are able to significantly alter cervical epithelial cell function, including the cellular processes regulating cervical remodeling, we wanted to investigate if the presence of *G. vaginalis* in the CV space of pregnant mice would result in preterm delivery. Intravaginal inoculation of *G. vaginalis* into the CV space resulted in a 100% PTB rate over a 96 h time period after initial exposure on E14 (**Figure 7A**). The sugar water control animals had no preterm deliveries over the same time period. To provide evidence that the intravaginal *G. vaginalis* inoculations resulted in the colonization of the CV space, we collected CVF, isolated gDNA, and measured *G. vaginalis* 16S rRNA 24 h after the first exposure. *G. vaginalis* 16s rRNA was significantly increased in the CVF of mice when compared to the sugar water controls (**Figure 7B**, *p* = 0.005).

## Discussion

This study provides novel evidence that bacteria common to the CV space play a significant role in regulating cervical epithelial cell function. We have shown that supernatants derived from *L. crispatus, L. iners*, and *G. vaginalis* have the ability to regulate the cervical epithelial barrier through multiple diverse mechanisms including cleavage of adherens junction proteins, altered inflammatory profiles, and differential epigenetic regulation through elevated miRNA expression. Additionally, this study shows that *L. crispatus*, a bacterium often associated with a “healthy” CV space, has the ability to protect the cervical epithelial barrier in the presence of either a general CV infection (LPS) or in the presence of bacteria associated with increased risk of BV, STI/STDs, or HIV (*G. vaginalis*). Interestingly, the protection conferred by *L. crispatus* bacteria-free supernatants in the presence of *G. vaginalis* seems to be mediated, at least in part, through miRNA-dependent mechanisms. Additionally, we show that *G. vaginalis* inoculated into the CV space of mice results in PTB, which suggests that the cervical dysfunction caused by *G. vaginalis* secreted factors are mechanistically linked to the cervical remodeling process. The results of this study support our hypothesis that the secreted factors derived from the CV microbiome play a significant role in regulating cervical epithelial cell function and that a dysbiotic state within the CV space could result in a disruption of the cervical epithelial barrier. The data from this study begin to ascribe the mechanisms linking secreted by-products from a specific CV microbiota to a direct cervical function – maintenance of the cervical epithelial barrier – and has implications for women’s health including not only adverse obstetrical outcomes (PTB) but also the acquisition of sexually transmitted diseases (STIs, HIV).

The cervical epithelial barrier (and its associated mucosal secretions) plays a significant role in both non-pregnant and pregnant states where it predominately acts to prevent the invasion of microbial pathogens. However, recent studies have suggested that the presence of known vaginal microbial pathogens such as *G. vaginalis* (BV) or *L. iners* (HIV, STIs) may have the ability to degrade the epithelial mucous layer resulting in biofilm production, cytolysis and inflammation of the cervical epithelial barrier (Gelber et al., 2008; Patterson et al., 2010; Rampersaud et al., 2011). The breakdown of the cervical epithelial barrier is a hypothesized critical step in the initiation of the cervical remodeling process as the disruption of the barrier allows for the influx of water into the cervical stroma resulting in cervical softening in preparation for delivery. Despite the associations between BV and/or *G. vaginalis* and adverse outcomes, there is a paucity of data investigating the biological mechanisms linking CV microbial pathogens to cervical epithelial barrier function and remodeling. Previous work from our laboratory, along with the data presented in this study, collectively demonstrated that colonization of the CV space with *G. vaginalis* leads to PTB through multiple mechanisms including disruption of the cervical epithelial barrier, augmentation of the cervical immune response, initiation of premature cervical remodeling, and modification of the biomechanical properties of the cervix (Sierra et al., 2018). Similarly, in this study, both *L. iners* and *G. vaginalis* bacteria-free supernatants significantly disrupt the ectocervical and endocervical epithelial barrier suggesting that these bacteria are secreting factors that have the ability to regulate cervical barrier integrity. We have previously attributed disruptions in the cervical epithelial barrier to an increase in the cleavage of E-cadherin, a member of the adherens junctional complex present on epithelial cells, by serine proteases, such as matrix metalloproteinases (MMPs) (Nold et al., 2012; Anton et al., 2017). The release of sECAD acts as a marker of decreased cell-to-cell adhesion within the epithelial barrier. While both *L. iners* and *G. vaginalis* bacteria-free supernatants increased sECAD release, only *L. iners* induced sECAD levels above those seen with the bacterial growth media alone. These results suggest that cleavage of E-cadherin might be an *L. iners*-specific mechanism of cervical epithelial breakdown that is not seen in the presence of *G. vaginalis*. Interestingly, a previous study has shown that increased abundance of *L. iners* is correlated with elevated extracellular metalloproteinase inducer (EMMPRIN) and MMP-8 concentrations in human cervical vaginal fluid (Witkin et al., 2013), providing further evidence for a role of CV bacteria in regulating the epithelial barrier. Importantly, it is also worth noting that the commensal *L. crispatus* bacteria-free supernatants did not alter the cervical epithelial barrier and had no effect on sECAD release, indicating, as hypothesized, that this bacterial strain may have beneficial effects on cervical epithelial barrier function.

The integrity of any epithelial barrier, independent of its biological location, is strongly dependent on host–microbiota interactions. Host cervical epithelial cells have the ability to create an “immune barrier” to protect against invading pathogens by producing cytokines and chemokines as part of the innate immune response as well as using mucous secretions and antimicrobial peptides as part of the mucosal immune response (Fichorova and Anderson, 1999; Herbst-Kralovetz et al., 2008; Radtke et al., 2012; Zaga-Clavellina et al., 2012; Doerflinger et al., 2014). The host epithelial innate immune response to both commensal and pathogenic bacteria has been shown to be mediated through toll-like receptor (TLR) signaling resulting in the upregulation of NF-kB and proinflammatory cytokines (Lebeis et al., 2007; Herbst-Kralovetz et al., 2008; Zheng et al., 2008; Abreu, 2010; Jung et al., 2012). However, the role of common CV bacteria in regulating the immune response by cervical epithelial cells remains mostly unknown. Data from this study show that the exposure of ectocervical epithelial cells to bacteria-free supernatants from *L. crispatus, L. iners*, and *G. vaginalis* elicited a widely varied and complex immune response. Perhaps not surprisingly and in agreement with previous studies (Zaga-Clavellina et al., 2012; Fichorova et al., 2013; Doerflinger et al., 2014; Gosmann et al., 2017), *L. iners* and *G. vaginalis*, bacteria commonly associated with BV and STI/HIV infections, induced a higher number of classical proinflammatory cytokines than the commensal bacteria, *L. crispatus*. However, the IL-8 and IL-6 levels (measured independently to validate our Luminex results) show no difference between *L. iners* bacteria-free supernatants and the background control media indicating that *L. iners*, a bacteria with intermediate effects on CV health (Petrova et al., 2017), may not significantly increase the host immune response. Interestingly, *L. crispatus* induced several proinflammatory cytokines, albeit to a lesser extent than that seen with *G. vaginalis*. While there is some disagreement in the existing literature (Rizzo et al., 2013; Doerflinger et al., 2014), this result has been shown previously as *L. crispatus* was able to increase NF-kB activity in vaginal epithelial cells (Fichorova et al., 2011). *L. crispatus* was also the only bacteria-free supernatant to increase IL-10 levels, and this agrees with previous studies showing a similar induction of IL-10 in HeLa cells (Rizzo et al., 2015). Assuming traditional inflammatory roles, this would suggest that *L. crispatus* has the ability to induce some anti-inflammatory effects within the CV space in the presence of both normal and dysbiotic conditions. The varied immune response by both typical commensal bacteria and bacteria associated with vaginal dysbiosis observed in our study provides evidence that the specific roles of individual bacterial species within the CV space are both complex and interconnected. While commensal bacteria are assumed to modulate the innate immune response in the CV space, some upregulation of this response could be seen as a compensatory mechanism by the host to also upregulate antimicrobial peptides, such as SLPI, which have been shown to be increased in the presence of LPS (an activator of the innate immune response) (Jin et al., 1997). The ambiguity and complexity of these immune results highlight the definitive need for future investigations into the role of both the cervical epithelial innate and mucosal immune response in the presence of a microbiota characteristic of normal and dysbiotic CV states. However, these data do provide evidence for a role of a host–microbiota immune response in the breakdown of the cervical epithelial barrier as we have previously shown that a bacterial by-product, such as LPS, can disrupt the cervical epithelial barrier through the upregulation of proinflammatory cytokines (Nold et al., 2012). Therefore, these studies suggest that the presence of a bacteria-induced immune response in the CV space has the potential to significantly alter cervical cell function.

The disruption of the cervical epithelial barrier has been linked to both inflammation-dependent and independent mechanisms, as we have previously shown that an upregulation of PTB-associated miRNAs, miR-143, and miR-145 in the cervix has the ability to breakdown the cervical epithelial barrier due to diverse mechanisms, including the regulation of cell adhesion, apoptosis, and cell proliferation (Elovitz et al., 2014; Anton et al., 2017). Multiple other studies have since found significant associations between cervical miRNA expression and the presence of specific bacteria and cytokine profiles as well as shorter gestational length (Sanders et al., 2015, 2017). Since relatively little is known about the epigenetic mechanisms linking the CV microbiota to alterations in cervical epithelial cell function, we investigated the expression of specific miRNAs after exposure to bacteria-free supernatants of *L. crispatus, L. iners*, and *G. vaginalis*. Interestingly, the upregulation of miR-143, miR-145, miR-146, miR-223, miR-148, and miR-15a by *G. vaginalis* bacteria-free supernatants and miR-193b, miR-146, and miR-223 by *L. iners* bacteria-free supernatants with no expression change in any of the measured miRNAs by *L. crispatus* shows the presence of a clear bacterial species-specific alteration in miRNA expression profiles. As one miRNA is known to regulate hundreds of downstream targets, it is of interest to investigate these miRNAs in a cluster as there will certainly be redundancy in their functions. As a cluster, these miRNAs have been previously associated with cervical epithelial barrier breakdown (Anton et al., 2017), PTB (Elovitz et al., 2014), short gestation (Sanders et al., 2015, 2017), BV-associated bacteria species (Sanders et al., 2017), or increased cervical inflammation (Sanders et al., 2017), suggesting that an alteration in cervical cell function is biologically plausible. We acknowledge that the exact downstream targets of these miRNAs within the CV space remain unknown and, therefore, the specific gene effects of altered miRNA expression are unclear; however, these studies do provide evidence that the secreted factors from bacteria associated with BV, STIs, or HIV (*L. iners* and *G. vaginalis*), but not commensal (*L. crispatus*), have the ability to epigenetically alter cervical cell function. Follow-up studies with in-depth investigation into each of these miRNAs and their downstream targets are warranted to identify the specific biological pathways regulating the effect of these miRNAs on cervical cell function. Additionally, future studies would be needed to ascertain the long-term effects of *L. iners* and *G. vaginalis* on the epithelial cell function within the CV space, as this study only investigated miRNA expression at one time point during bacteria-free supernatant exposure (48 h post-exposure). Taken together, these data provide evidence of a role for secreted factors derived from the CV microbiome in the induction of epigenetic modification of cervical epithelial cells in the presence of a dysbiotic state.

*Lactobacillus crispatus* is most often associated with a “healthy” vaginal state as the presence and/or dominance of *L. crispatus* has been shown to be indicative of a lower risk of BV (Dols et al., 2016). *L. crispatus* has several antibacterial properties, making it a highly investigated option as a vaginal probiotic, including lowering vaginal pH by the production of the metabolites hydrogen peroxide and lactic acid and by the upregulation of antimicrobial molecules such as bacteriocins and antimicrobial peptides (Reid and Burton, 2002; Servin, 2004; Spurbeck and Arvidson, 2011). Our study provides evidence that *L. crispatus* confers significant protection to the cervical epithelial barrier against a CV inflammogen resulting from the presence of increased LPS (derived from *E. coli*) or bacteria associated with an increased risk of CV disease (such as *G. vaginalis*). The results of this study show that *L. crispatus* protects the cervical epithelial barrier in the presence of an inflammatory stimulus; however, the mechanisms regulating this interaction will need further investigation as these experiments are not able to identify if *L. crispatus* bacteria-free supernatants have the ability to block LPS binding to the TLR4 receptor or activate the host immune response to prevent the inflammatory insult. While there are previous studies that have shown a protective effect of *L. crispatus* in the face of *Prevotella bivia* and *G. vaginalis* (BV-associated bacteria) (Atassi et al., 2006), *E. coli* (Ghartey et al., 2014), HIV acquisition (Gosmann et al., 2017), and cervical cancer (human papillomavirus oncogenes) (Wang et al., 2017), none have investigated the direct protective effects of *L. crispatus* on either the cervical epithelial barrier or the biological mechanisms involved in this effect. In the presence of *L. crispatus* bacteria-free supernatants, the *G. vaginalis*-induced increase in miRNA expression was robustly reduced, suggesting that *L. crispatus* bacteria-free supernatants are able to mitigate the *G. vaginalis*-mediated disruption of the cervical epithelial barrier through a reduction in miRNA expression. While reduced miRNA expression is undoubtedly not the only mechanism contributing to the protection of the cervical epithelial barrier by *L. crispatus*, these results do suggest that epigenetic regulation of cervical cell function could play a significant role in upholding barrier integrity. This result has significant implications not only for adverse obstetrical outcomes such as PTB where the breakdown of the cervical epithelial barrier could initiate the cervical remodeling process early in gestation but also in cases of sexually transmitted diseases such as HIV where the presence of *L. crispatus* might be able to prevent or mitigate HIV from crossing the cervical epithelial barrier and gaining access to the cervical stroma (and the underlying susceptible target cells) thus reducing the infection rate (Bomsel, 1997; Hocini and Bomsel, 1999). Interestingly, a previous study has shown that women with a high-diversity and low-*Lactobacillus*-abundant vaginal microbiome are at a higher risk for HIV acquisition, where the authors hypothesized this to be due to the presence of pathogenic bacteria-induced increases in LPS, which recruits HIV target cells (CD4+ and Th17) to the vaginal mucosa (Gosmann et al., 2017). Additionally, studies have found that HIV has the ability to disrupt and traverse the epithelial barrier by downregulating tight junction proteins as well as increasing proinflammatory cytokine production both by the virus itself and when HIV is present in combination with *Chlamydia trachomatis* infection (Nazli et al., 2010; Buckner et al., 2016). Taken together, the results of our study suggest that the disruption of the cervical epithelial barrier by a dysbiotic CV microbiome and the conferred protection by *L. crispatus* could play a significant role in preventing prevalent OB/GYN pathologies in both pregnant and non-pregnant states.

While our *in vitro* studies have provided evidence that bacteria associated with unhealthy vaginal states, *L. iners* and *G. vaginalis*, result in significant cervical epithelial dysfunction, it remained unknown if activation of these biological processes were sufficient to induce PTB. Our animal model successfully recapitulated the human vaginal microbiota by colonizing the CV space with *G. vaginalis* that resulted in 100% PTB. These data support the hypothesis that *G. vaginalis* colonization disrupts the cervical epithelial barrier, leading to premature cervical remodeling and subsequent PTB. While a non-pregnant mouse model demonstrated that *G. vaginalis* could colonize the CV space (Gilbert et al., 2013), this study did not investigate the effects of *G. vaginalis* colonization on cervical cell function or cervical barrier integrity. The animal model described in this manuscript is the first report of a pregnant mouse model with intravaginal colonization by *G. vaginalis* that results in significant PTB. Importantly, we demonstrate that *G. vaginalis* bacterial colonization of the CV space leads to an adverse birth outcome. These data add to our prior report that *G. vaginalis* colonization of the CV space results in the initiation of cervical remodeling in CD1 mice (Sierra et al., 2018). Lower levels of PTB were reported in our previous study compared to those presented here. This difference is most notably attributed to the different mouse strains used in the two studies (CD1 vs. C57/B6) as CD1 mice are an outbred strain that inherently results in more experimental variation. In addition to these data, another recently published study demonstrated that the colonization of Group B *Streptococcus agalactiae* in the CV space of pregnant C57/B6 mice resulted in the disruption of the vaginal epithelial barrier resulting in PTB (although the exact PTB rates were not reported) or fetal death *in utero* (Vornhagen et al., 2018). Previous studies using non-pregnant mice (either CD1 or C57/B6) supplemented with hormones (17β-estradiol) have also shown successful colonization of the mouse CV space with bacteria associated with adverse health outcomes. These studies used a variety of pathogens including *G. vaginalis* (Gilbert et al., 2013), Group B *Streptococcus agalactiae* (Patras et al., 2015; Vornhagen et al., 2018), and *Candida albicans* (Pietrella et al., 2011). Similar to our animal model, these studies used comparable intravaginal inoculation methods and bacterial loads (CFUs 10^7^–10^9^). Since hormone levels (estrogen and progesterone) are already elevated in the pregnant mouse, exogenous administration of hormones was not required in our model, unlike the non-pregnant models. The creation of a pregnant mouse model with intravaginal *G. vaginalis* colonization will provide a novel system in which to further study the effects of *G. vaginalis* on both vaginal and cervical epithelial dysfunction as well as the interactions between bacterial and host immunity. We acknowledge that the colonization of the CV space with a single bacterial species does not represent the human CV microbial community as a whole and limits the study of the interaction between bacterial species on host cervical function. However, this animal model does provide clear evidence of a biological mechanism linking a specific bacterium, *G. vaginalis*, to PTB.

Overall, the results from this study show that bacteria common to the CV space, *L. crispatus, L. iners*, and *G. vaginalis*, play a significant role in regulating cervical epithelial cell function in both normal and dysbiotic states. Secreted factors produced by bacteria associated with increased risk of disease (BV, STIs, and HIV), *L. iners* and *G. vaginalis*, disrupt the cervical epithelial barrier by diverse mechanisms including the cleavage of adherens junction proteins, the upregulation of proinflammatory immune mediators, and increased miRNA expression. *G. vaginalis* colonization within the CV space of mice resulted in PTB providing further evidence for a role of *G. vaginalis* in cervical epithelial cell dysfunction, which leads to a disruption of the cervical epithelial barrier and the initiation of cervical remodeling. Protection of the cervical epithelial barrier conferred by *L. crispatus* secreted factors in the presence of infection-inducing bacteria (or bacterial by-products) provides evidence for a beneficial effect of *L. crispatus* to women who may be at a higher risk for PTB. Future studies are warranted to investigate if therapeutics that promote an *L. crispatus* dominate CV state would be useful in the prevention of STI acquisition and/or PTB by preventing cervical epithelial dysfunction.

## Author Contributions

LA wrote the manuscript, created the figures, and analyzed the data. LA, AB, and ME conceived and designed the experiments. LA, AD, L-JS, GB, and LH performed the experiments. All authors contributed to manuscript revision and approved the final manuscript.

## Conflict of Interest Statement

The authors declare that the research was conducted in the absence of any commercial or financial relationships that could be construed as a potential conflict of interest.
