# Supplementary material for: Common Cervicovaginal Microbial Supernatants Alter Cervical Epithelial Function: Mechanisms by Which Lactobacillus crispatus Contributes to Cervical Health
Source: Front Microbiol. 2018 Oct 8;9:2181. doi: 10.3389/fmicb.2018.02181 (PMC6186799; doi:10.3389/fmicb.2018.02181)
Supplement: Supplementary file 1 [file Data_Sheet_1.DOCX]

Common cervicovaginal microbial supernatants alter cervical epithelial function: mechanisms by which *L. crispatus* contributes to cervical health

**Authors:** Lauren Anton^1*^, Ann DeVine^1^, Luz-Jeannette Sierra^1^, Laura Heiser^1^, Amy G. Brown^1^, Michal A. Elovitz^1^

***Corresponding Author:**

Lauren Anton

E-mail: [lanton@pennmedicine.upenn.edu](mailto:lanton@pennmedicine.upenn.edu)

| **Bacterial Species** | **Range of Bacteria Abundance (CFU/ml)** |
| --- | --- |
| *L. crispatus* | 2x10^7^ - 13x10^7^ |
| *L. iners* | 3x10^10^ - 11x10^10^ |
| *G. vaginalis* | 17.5 X10^7^ - 21.8x10^7^ |

**Supplemental Table 1. Bacterial abundance used for study experiments.** The range of bacterial abundance for *L. crispatus*, *L. iners* and *G. vaginalis* utilized to make the bacteria-free supernatants used in the experiments in this study.

| Analyte | Detectable in Ectocervical Cells? | Minimum Detectable Concentration (pg/ml) |
| --- | --- | --- |
| EGF | Present at high levels in basal growth media |  |
| FGF-2 | No | <12.82 |
| Eotaxin | No | <2.96 |
| Fractalkine | No | <3.40 |
| IFNa2 | No | <3.94 |
| IFNr | No | <0.80 |
| MCP-3 | No | <2.95 |
| IL-12P70 | No | <2.87 |
| IL-13 | No | <0.88 |
| sCD40L | No | <3.96 |
| IL-17A | No | <0.00 |
| IL-9 | No | <2.91 |
| IL-2 | No | <1.00 |
| IL-3 | No | <2.86 |
| IL-4 | No | <3.09 |
| IL-5 | No | <2.61 |
| MCP-1 | No | <2.09 |
| MIP-1a | No | <5.13 |
| MIP-1b | No | <0.75 |
| TNFb | No | <0.30 |

**Supplemental Table 2. Non-detectable cytokines/chemokines by Luminex Assay.** Cytokines/chemokines included in the Luminex Assay that were non-detectable in either the *L. crispatus*, *L, iners* or *G. vaginalis* bacteria-free supernatant treated culture media or the non-treated culture media of ectocervical epithelial cells after 48 hours of exposure.

|  | **Ectocervical Cells** | | | | | | | | | | | |
| --- | --- | --- | --- | --- | --- | --- | --- | --- | --- | --- | --- | --- |
|  | L. crispatus | | L. crispatus Media | | L. iners | | L. iners Media | | G. vaginalis | | G. vaginalis Media | |
| **Target** | Fold Change | P value | Fold Change | P value | Fold Change | P value | Fold Change | P value | Fold Change | P value | Fold Change | P value |
| **miR-21** | -1.19 | 0.8206 | -1.24 | 0.7499 | 3.26 | 0.0050 | 2.71 | 0.0190 | -1.22 | 0.5694 | -1.17 | 0.6420 |
| **miR-142** | 1.03 | 0.9631 | -1.83 | 0.0088 | 1.32 | 0.5887 | -2.02 | 0.3126 | 1.03 | 0.9971 | 1.08 | 0.9770 |
| **miR-494** | 1.22 | 0.3421 | -1.27 | 0.5678 | 1.82 | 0.0597 | 1.08 | 0.9536 | 1.62 | 0.2942 | 1.73 | 0.1904 |
| **miR-30e** | -1.09 | 0.7500 | -1.44 | 0.0851 | 1.38 | 0.3819 | -1.38 | 0.5663 | 1.08 | 0.8809 | -1.02 | 0.9958 |

**Supplemental Table 3. miRNAs showing no significant alteration in expression after exposure to bacteria-free supernatants.** A list of miRNAs whose expression remained significantly unchanged after exposure to bacterial-free supernatants from *L. crispatus*, *L. iners* and *G. vaginalis*.
